# Estimating potential palliative care needs for residential aged care: A population‐based retrospective cohort study

**DOI:** 10.1111/ajag.13345

**Published:** 2024-06-24

**Authors:** Greer B. Humphrey, Maria C. Inacio, Catherine Lang, Owen F. Churches, Janet K. Sluggett, Helena Williams, Deidre D. Morgan, Timothy H. M. To, Andrew Kellie, Steve Wesselingh, Gillian E. Caughey

**Affiliations:** ^1^ Health Translation South Australia South Australian Health and Medical Research Institute Adelaide South Australia Australia; ^2^ Registry of Senior Australians (ROSA) South Australian Health and Medical Research Institute Adelaide South Australia Australia; ^3^ UniSA Allied Health and Human Performance University of South Australia Adelaide South Australia Australia; ^4^ Silver Chain Group Limited Adelaide South Australia Australia; ^5^ Palliative and Supportive Services, College of Nursing and Health Sciences Flinders University Adelaide South Australia Australia; ^6^ Research Centre for Palliative Care, Death and Dying Flinders University Adelaide South Australia Australia; ^7^ Division of Rehabilitation, Aged Care and Palliative Care Flinders Medical Centre Bedford Park South Australia Australia; ^8^ Flinders University College of Nursing and Health Sciences Bedford Park South Australia Australia; ^9^ East Adelaide Healthcare Newton South Australia Australia

**Keywords:** long‐term care, palliative care, residential facilities

## Abstract

**Objective:**

Population‐based data on the required needs for palliative care in residential aged care have been highlighted as a key information gap. This study aimed to provide a comprehensive estimate of palliative care needs among Australia's residential aged care population using a validated algorithm based on causes of death.

**Methods:**

A population‐based retrospective cohort study was conducted using data from the Registry of Senior Australians of non‐Indigenous residents of residential aged care services in New South Wales, Victoria, and South Australia aged older than 65 years, who died between 2016 and 2017 (*n* = 71,677). An internationally validated algorithm was used to estimate and characterise potential palliative care needs based on causes of death. This estimate was compared to palliative care needs identified from funding‐based care needs assessment data.

**Results:**

Ninety two per cent (*n* = 65,949) were estimated to have had potential palliative care needs prior to their death. Of these, 19% (*n* = 12,467) were assigned an end‐of‐life trajectory related to cancer, 61% (*n* = 40,511) to organ failure and 20% (*n* = 12,971) to frailty and dementia. By comparison, only 6% (*n* = 4430) of residents were assessed as needing palliative care by the funding‐based care needs assessment.

**Conclusions:**

Over 90% of individuals dying in residential aged care may have benefited from a palliative approach to care. This need is substantially underestimated by the funding‐based care needs assessment, which utilises a narrow definition of palliative care when death is imminent. There is a clear imperative to distinguish between palliative and end‐of‐life care needs within residential aged care to ensure appropriate and equitable access to palliative care.

**Practice Impact**

Over 90% of individuals in residential aged care may have benefited from a palliative approach to care. This estimate likely reflects the true need for palliative care in this population and importantly identified potential unmet need.

**Policy Impact**

There is a clear imperative to distinguish between palliative and end‐of‐life care needs within the residential aged care sector to ensure appropriate and equitable access to palliative care.

## 1 INTRODUCTION

Residential aged care plays an increasing role in caring for older people towards the end of life and is commonly a person's final place of residence before they die.^1^ Older people enter residential aged care when their care needs become too complex to manage at home. The majority have multimorbidity, around half have dementia and many have a life‐limiting chronic disease, with declining function and well‐being.^2^, ^3^ A palliative approach to care that focusses on quality of life and managing symptoms to support individuals to live as comfortably as possible is beneficial throughout their illness and not just when death is imminent.^4^

While Australia has been recognised as a world leader in the provision of palliative care,^5^ the recent Royal Commission into Aged Care Quality and Safety reported that too few people in aged care receive evidence‐based palliative care and identified the provision of palliative care within the residential aged care sector as a priority for immediate attention.^6^ Similar concerns have also been raised in other countries.^7^, ^8^ A barrier to the integration of a palliative approach to care in residential aged care is the misconception that palliative care is synonymous with end‐of‐life care.^9^ However, these concepts are distinct. Palliative care aims to prevent and relieve suffering for individuals with a life‐limiting illness that may be needed early and episodically throughout the course of a life‐limiting illness.^10^ In contrast, end‐of‐life care typically refers to the 12 months prior to death; although in practice, it is often used to describe care provided in the last days of life.^11^ In Australia, an assessment of core aged care needs, including palliative care, is conducted at entry into residential aged care or if care needs change, as a basis for allocating government funding.^12^ However, the definition of palliative care under this care needs assessment is restricted to the provision of end‐of‐life care during the last week or days of life.^12^, ^13^ This narrow view of palliative care means that data on palliative care needs collected from this assessment underestimate the true need for palliative care in the residential aged care population and robust national data on the need for palliative care in the aged care setting has been highlighted as a key information gap.^13^, ^14^ With no nationally consistent data collection or methodology to quantify need for palliative care in Australia,^14^ there is limited insight regarding palliative care needs (or unmet needs) and service requirements within residential aged care. A more complete picture of palliative care needs within the sector is essential to inform the appropriate provision of equitable, high‐quality palliative care in residential aged care.

The aim of this study was to estimate the potential need for palliative care, including both generalist and specialist palliative care among Australia's residential aged care population using a validated population‐based algorithm based on cause of death diagnoses. We also examined the identification of residents requiring palliative care using the funding‐based care needs assessment in residential aged care and examined factors associated with the assessment of requiring palliative care.

## 2 METHODS

### 2.1 Study design and data sources

A population‐based retrospective observational study was conducted using the Registry of Senior Australians (ROSA) National Historical Cohort, which includes de‐identified linked aged care and health‐care data.^3^ This study utilised the Australian Institute of Health and Welfare National Aged Care Data Clearinghouse data included in ROSA, specifically the: Residential Aged Care Episodes, National Death Index (NDI), Aged Care Assessment Program (aged care eligibility assessment) and the Aged Care Funding Instrument (funding‐based care needs assessment).^3^ The NDI data set provides dates and causes of death, coded using the International Classification of Diseases, 10th revision, Australian modification (ICD‐10‐AM).^3^

This study had ethics approval from University of South Australia (Ref: 200489) and Australian Institute of Health and Welfare (Ref: EO2018/1/418) Human Research Ethics Committees.

### 2.2 Study cohort

The study cohort included all non‐Indigenous individuals aged older than 65 years, who died between 1 January 2016 and 31 December 2017 and who were living in permanent residential aged care in South Australia, New South Wales or Victoria in the 3 years prior to their death. The study cohort was restricted to individuals in these three states, which had complete data capture within the ROSA National Historical Cohort at the time of data analysis. Individuals with unknown or unreported underlying cause of death were excluded from the analysis (*n* = 11), as were individuals who did not have a funding‐based care needs assessment (*n* = 589).

### 2.3 Estimation of palliative care needs

A method for estimating population‐based palliative care need using cause of death (from NDI data) developed by the French National Observatory on End‐of‐Life Care (ONFV)^15^ was used to identify individuals who could have benefited from receiving palliative care prior to death. This validated approach compares individuals' cause of death data to a list of chronic conditions (classified using ICD‐10 diagnosis codes) identified by expert clinicians as those most relevant for palliative care.^16^ When compared with similar methodologies for estimating population‐based palliative care needs,^17^, ^18^ the ONFV approach most closely aligns with the World Health Organization definition of diseases requiring palliative care for adults.^10^, ^16^ A further advantage of the ONFV approach is that conditions are classified into three end‐of‐life trajectories: (1) Cancer, defined by a short decline with a usually clear terminal phase; (2) Organ failure, defined by gradual decline, punctuated by acute episodes of deterioration and sometimes sudden and unexpected death; and (3) Frailty and dementia, defined by a gradual and prolonged decline.^15^, ^19^

Using the ONFV, residents' palliative care needs were estimated and classified according to end‐of‐life trajectory using multiple cause of death data (reported using ICD‐10‐AM diagnosis codes), including the principal (underlying) cause of death as well as the associated (contributing) causes of death. In instances where the recorded causes of death included ICD‐10‐AM codes across multiple end‐of‐life trajectories, individuals were assigned a unique end‐of‐life trajectory based on the predefined hierarchy: cancer > organ failure > frailty and dementia.^20^ This is based on the premise that cancer would be the most dominant and rapidly progressing trajectory, followed by organ failure and then frailty and dementia (with the latter defined by a gradual and prolonged decline).^20^ For example, while there is a high prevalence of frailty and dementia in the residential aged care population,^21^, ^22^ individuals were only assigned this end‐of‐life trajectory if their causes of death, both principal and associated, did not also classify them as having the cancer or organ failure trajectory. Unclassified individuals were considered unlikely to have palliative care needs, dying from causes such as pneumonia, septicaemia, accidental falls and acute myocardial infarction. The specific ICD‐10‐AM codes and groupings are provided in Table S1.

### 2.4 Funding‐based assessment of palliative care needs

Palliative care needs were also examined using the funding‐based aged care needs assessment data. Within this assessment, the definition of palliative care needs includes residents requiring end‐of‐life care in the last week or days of life involving ‘very intensive clinical nursing and/or complex pain management in the residential care setting’ (p. 42).^12^ During the study period, this assessment was mandatory within 2 months of admission to residential aged care, and reassessments could be conducted after a 12‐month period or within 12 months if there was a significant change in care needs. In this study, data from the latest assessment before death were used to identify individuals appraised as needing palliative care.

### 2.5 Covariates

Characteristics of the cohort were examined including age at death, sex, marital status at the time of aged care assessment (partnered, unpartnered), country of birth (Australia, overseas), preferred language (English, other), remoteness of residence (using Accessibility / Remoteness Index of Australia), socio‐economic status (based on residential postcode mapped to quintiles of the Index of Relative Socioeconomic Disadvantage), length of stay in residential aged care prior to death (0–3, 3–6, 6–12, >12 months) and causes of death. Both underlying and contributing causes of death were categorised into non‐mutually exclusive disease groups including the following common chronic and life‐limiting conditions: dementia, coronary heart disease, cerebrovascular disease, heart failure, cancer (any malignant), kidney failure, diabetes, chronic obstructive pulmonary disease (COPD), Parkinson's disease, chronic respiratory failure, liver disease, motor neuron disease and multiple sclerosis.

### 2.6 Statistical analysis

Descriptive statistics were used to describe the sociodemographic characteristics of the study cohort and causes of death. Multivariate logistic regression analysis was also used to examine the adjusted odds ratios (aORs) and 95% confidence intervals (CIs) of sociodemographic factors and recorded causes of death associated with being assessed as needing palliative care using the funding‐based care needs assessment. A stepwise variable selection approach was used, with the Akaike information criterion used for model selection, and only complete cases were analysed (<2% of cases had missing data). Reporting of this in this study is done in accordance with the STrengthening the Reporting of OBservational Studies in Epidemiology (STROBE) statement.^23^ All statistical analyses were conducted using R v4.0 (R Core Team 2020, Vienna, Austria; www.R‐project.org).

## 3 RESULTS

Of the 71,677 residents who died between 2016 and 2017 and were included in the study, 62% (*n* = 44,157) were female, median age at death was 88 years old (interquartile range [IQR] 83–93) and approximately 70% (*n* = 49,840) lived in a major city (Table 1). Within the first three months following entry to residential aged care, 11% (*n* = 7173) had died, and 30% (*n* = 21,592 by 12 months). Dementia was the most common recorded underlying or contributory cause of death (41%, *n* = 29,534), followed by coronary heart disease (23%, *n* = 16,730), cerebrovascular disease (19%, *n* = 13,510), heart failure (19%, *n* = 13,240) and cancer (16%, *n* = 11,661) (Table 2). Approximately 65% (*n* = 46,536) had at least two aged care needs assessments between entry to residential aged care and death and less than a quarter (23%, *n* = 16,656) had an assessment within 3 months of death.

**TABLE 1:** Characteristics of overall study cohort (*n* = 71,677), estimation of palliative care needs by type of end‐of‐life trajectory and funding‐based palliative care assessment.

	Overall study cohort^a^, *n* = 71,677	Estimated need for palliative care (ONFV)^15^ *n* = 65,949 (92%)	Specific end‐of‐life trajectories, *N* (%)^c^			Funding‐based palliative care assessment, *n* = 4430 (6%)
			Cancer, *n* = 12,467 (19%)	Organ failure, *n* = 40,511 (61%)	Frailty and dementia, *n* = 12,971 (20%)	
Gender						
Female	44,157 (62)	40,363 (61)	6346 (51)	25,434 (63)	8583 (66)	2482 (56)
Male	27,520 (38)	25,586 (39)	6235 (49)	15,077 (37)	4388 (34)	1948 (44)
Age at death						
Median (IQR)	88 (83, 93)	88 (83, 92)	87 (81, 91)	89 (84, 93)	88 (83,93)	86 (81, 91)
65–74 years	4555 (6)	4276 (7)	1208 (10)	2176 (5)	892 (7)	477 (11)
75–84 years	16,868 (24)	15,863 (24)	3596 (29)	8947 (22)	3320 (26)	1310 (30)
85–94 years	39,270 (55)	36,073 (55)	6454 (52)	22,869 (57)	6750 (52)	2179 (49)
≥95 years	10,984 (15)	9737 (15)	1209 (10)	6519 (16)	2009 (16)	464 (11)
Remoteness						
Major City	49,840 (70)	45,731 (69)	8477 (68)	28,024 (69)	9230 (71)	2677 (60)
Rural or remote	21,083 (29)	19,537 (30)	3909 (31.4)	12,061 (29.8)	3567 (27.5)	1715 (38.7)
Marital status						
Partnered	26,122 (36)	24,305 (37)	4737 (38)	14,204 (35)	5364 (41)	1740 (39)
Unpartnered	45,554 (64)	41,643 (63)	7730 (62)	26,306 (65)	7607 (59)	2690 (61)
Country of birth						
Australia	48,985 (68)	45,066 (68)	8683 (70)	27,613 (68)	8770 (68)	3136 (71)
Overseas	22,339 (31)	20,551 (31)	3705 (30)	12,729 (31)	4117 (32)	1271 (29)
Preferred language						
English	63,420 (89)	58,411 (89)	11,287 (91)	35,673 (88)	11,451 (88)	4055 (92)
Other	8125 (11)	7420 (11)	1151 (9)	4774 (12)	1495 (12)	365 (8)
Socioeconomic status						
1 (most disadvantaged)	12,571 (18)	11,577 (18)	2301 (19)	7285 (18)	1991 (15)	776 (18)
2	14,339 (20)	13,287 (20)	2708 (21)	8167 (20)	2457 (19)	994 (22)
3	13,561 (19)	12,525 (19)	2333 (19)	7736 (19)	2456 (19)	839 (19)
4	12,257 (17)	11,243 (17)	2106 (17)	6920 (17)	2217 (17)	736 (17)
5 (least disadvantaged)	18,194 (25)	16,636 (25)	2983 (24)	9977 (25)	3676 (28)	1047 (24)

Abbreviations: ACFI, aged care funding instrument; IQR, interquartile range; ONFV, Observatoire National de la Fin de Vie (French National Observatory on End‐of‐Life Care).^15^

^a^ Missing from total cohort: Country of birth *n* = 353 (1%); Preferred language *n* = 132 (0%); Remoteness *n* = 754 (1%), Socioeconomic status *n* = 755 (1%).

**TABLE 2:** Causes of death and length of stay in residential aged care in the overall cohort and those assessed as needing palliative care using the funding‐based aged care assessment.

	Overall study cohort	Funding‐based palliative care assessment
	*n* = 71,677	*n* = 4430 (6%)
	*n* (%)	*n* (%)
Causes of death^a^		
Dementia	29,534 (41)	1242 (28)
Coronary heart disease	16,730 (23)	854 (19)
Cerebrovascular disease	13,510 (19)	641 (15)
Heart failure	13,240 (19)	741 (17)
Cancer (any malignant)	11,661 (16)	1790 (40)
Kidney failure	9400 (13)	543 (12)
Diabetes	9204 (13)	505 (11)
COPD	6767 (9)	423 (10)
Parkinson's disease	3172 (4)	120 (3)
Chronic respiratory failure	3101 (4)	181 (4)
Liver disease	692 (1)	92 (2)
Motor neuron disease	238 (0)	30 (1)
Multiple sclerosis	141 (0)	5 (0)
Length of stay in residential aged care before death		
0–3 months	8086 (11)	2265 (51)
3–6 months	5430 (8)	297 (5)
6–12 months	8076 (11)	333 (4)
>12 months	50,085 (70)	1535 (35)
Number of funding‐based aged care assessments		
1	25,141 (35)	2367 (53)
2+	46,536 (65)	2063 (47)
Assessment within 3 months of death	16,656 (23)	3868 (87)

Abbreviations: COPD, Chronic obstructive pulmonary disease.

^a^ May add up to more than 100% (underlying and associated causes of death).

Using the ONFV estimation approach, it was identified that 92% (*n* = 65,949) of the 71,677 residents who died in 2016–2017 potentially had palliative care needs. Examination by end‐of‐life trajectory resulted in the following classification of these individuals: 19% (*n* = 12,467) cancer, 61% (*n* = 40,511) organ failure and 20% (*n* = 12,971) frailty and dementia (Table 1). Some differences in the characteristics of the groups were observed; residents assigned the cancer end‐of‐life trajectory were younger (median age at death 87 years) and more likely to live in areas with greater socio‐economic disadvantage (40% in the two most disadvantaged quintiles). Residents assigned the organ failure trajectory were the oldest with a median age at death of 89 years and 73% of residents aged older than 85 years, while those assigned the frailty and dementia trajectory were more likely to be female (66%), partnered (41%) and living in the highest socio‐economic quintile area (28% in quintile 5 [least disadvantaged]) (Table 1).

Using the funding‐based aged care assessment data, 6% (*n* = 4430) of the study cohort were assessed as needing palliative care (Table 1). Of those, 40% (*n* = 1790) died from cancer and 51% (*n* = 2265) died within 3 months from residential aged care entry. Over half (*n* = 2367) had one funding‐based aged care needs assessment between entry and death, and 87% (*n* = 3868) were assessed 3 months prior to death (Table 2).

For those who entered residential aged care more than 3 months before death, the likelihood of being assessed as needing palliative care using the funding‐based aged care assessment were at least 85% lower than those who entered residential aged care within 3 months of death (length of stay 3–6 months vs 0–3 months, aOR 0.15, 95% CIs 0.14–0.18) (Table 3). However, adjusting for length of stay prior to death and other potential confounding, increasing levels of socio‐economic advantage (socio‐economic status quintile 5 [least disadvantage]) compared to socio‐economic status quintile 1 (most disadvantage, aOR 1.32, 95% CI 1.18–1.48) and living in a remote or rural area compared to a major city (aOR 1.64, 95% CI 1.52–1.78) were independently associated with increased likelihood of being assessed as having palliative care needs using the funding‐based aged care assessment. Increasing age was negatively associated with being assessed as needing palliative care (aOR .88, 95% CI .84–.92 per 10‐year increase in age at death), as was being male (aOR .91, 95% CI .85–.97) and speaking a language other than English (aOR .89, 95% CI .79–1.00). Residents with specific conditions recorded as a cause of death including cancer (aOR 2.49, 95% CI 2.31–2.68) and liver disease (aOR 1.61, 95% CI 1.25–2.06) were more likely to be appraised as needing palliative care. By comparison, residents with dementia (aOR .92, 95% CI .85–.99), coronary heart disease (aOR .87, 95% CI .80–.95), cerebrovascular disease (aOR .90, 95% CI .82–.99), heart failure (aOR .91, 95% CI .83–.99), diabetes (aOR .84, 95% CI .76–.94), Parkinson's disease (aOR .72, 95% CI .59–.87) or chronic respiratory failure (aOR .84, 95% CI .71–.99) as a cause of death were less likely to be assessed as having palliative care needs (Table 3).

**TABLE 3:** Factors associated with being assessed as needing palliative care using the funding‐based aged care assessment.

	aOR (95% CI)	*p*‐Value
Gender		
Female	1.00 (ref)	
Male	.91 (.85–.97)	.006
Age at death		
Per 10 year increase above 65 years	.88 (.84–.92)	<.001
Remoteness		
Major City	1.00 (ref)	
Rural or remote	1.64 (1.51–1.77)	<.001
Language		
English	1.00 (ref)	
Other	.89 (.79–1.00)	.06
Socioeconomic status		
1 (most disadvantaged)	1.00 (ref)	
2	1.17 (1.05–1.30)	.004
3	1.15 (1.03–1.28)	.02
4	1.28 (1.14–1.45)	<.001
5 (least disadvantaged)	1.33 (1.19–1.49)	<.001
Cause of death (yes vs no)		
Dementia	.92 (.85–.99)	.03
Coronary heart disease	.87 (.80–.95)	.001
Cerebrovascular disease	.90 (.82–.99)	.02
Heart failure	.91 (.83–.99)	.03
Cancer (any malignant)	2.49 (2.31–2.68)	<.001
Diabetes	.84 (.76–.94)	.001
COPD	.90 (.80–1.01)	.07
Parkinson's disease	.72 (.59–.87)	.001
Chronic respiratory failure	.84 (.71–.99)	.04
Liver disease	1.61 (1.25–2.06)	<.001
Motor neuron disease	1.46 (.94–2.18)	.08
Multiple sclerosis	.50 (.17–1.18)	.2
Time from residential care entry to death		
0–3 months	1.00 (ref)	
3–6 months	.15 (.14–.18)	<.001
6–12 months	.12 (.11–.14)	<.001
>12 months	.10 (.09–.11)	<.001

Abbreviations: aOR, adjusted odds ratio; CI, confidence interval; COPD, Chronic obstructive pulmonary disorder.

## 4 DISCUSSION

### 4.1 Main findings

This large population‐based study of over 71,000 individuals in residential aged care identified that 92% may have benefited from a palliative approach to care. This estimate, using an internationally validated algorithm based on disease diagnoses, likely reflects the true need for palliative care in this population. With 56,000 individuals dying as residents of residential aged care in Australia in 2020–2021,^24^ this equates to an estimated 51,500 individuals who would likely have benefited from a palliative care approach focussed on symptom management and quality of life.

Over the next two decades, projections estimate an unprecedented increase in the number of individuals in high‐income countries requiring palliative care, attributed to increasing deaths from chronic diseases namely dementia and cancer, together with increasing overall deaths.^25^ The demographic profile of aged care residents is also changing, with individuals entering residential aged care at an older age with increasingly complex care requirements.^26^, ^27^ Approximately 80% of residents die in residential aged care^1^ and is imperative to properly recognise and measure the need for palliative care within the residential aged care sector to support high‐quality care and a workforce skilled in palliative care delivery.

### 4.2 Current inadequate assessment of palliative care needs

Only 6% of residents were assessed as requiring palliative care using the funding‐based aged care assessment in accordance with its narrow definition of palliative care in the last week or days of life.^12^, ^13^ In October 2022, a new funding‐based assessment tool was implemented, the Australian National Aged Care Classification, with palliative care needs identified on the basis of a life expectancy of 3 months or less.^28^ However, this new definition is still reflective of end of life care (which is a specific phase of palliative care)^4^ and with fewer than 12% of individuals dying within 3 months of entry to residential aged care, this will still underestimate true palliative care needs of residents. Sociodemographic factors including older age, lower socio‐economic status and speaking a language other than English were found to be negatively associated with being assessed as requiring palliative care, highlighting potential structural barriers. It has also been reported that residential aged care services may not undertake an additional funding‐based care needs assessment if a resident becomes end of life, if the resident was already on the maximum subsidy payable and would therefore not increase in such cases.^29^ It is important to acknowledge that ascertainment of palliative care needs derived from the funding‐based assessment may not be concordant with the actual provision of palliative care to residents and is likely an underestimate of the actual delivery of palliative care in residential aged care. Nevertheless, analyses conducted by the Australian Institute of Health and Welfare indicated that of the people who died in Australian residential aged care in 2016–2017, only 18% received some form of palliative care service in the preceding year, including being prescribed palliative care medications, being seen by a palliative care specialist, or being admitted to hospital for palliative care.^1^

These findings demonstrate the inadequacy of the past and new national funding‐based aged care needs assessment data to provide insight into palliative care needs and workforce requirements, or plan for future demands and equitable access of palliative care.^8^, ^25^ The findings also highlight that palliative care in the residential aged care sector is likely to be largely under resourced, given the substantial mismatch between those estimated to have palliative care needs using the population‐based approach in the current study compared to those appraised as requiring funding for palliative care. There is a clear imperative to distinguish between palliative and end‐of‐life care needs for individuals entering residential aged care with life‐limiting illnesses and allowing the existing funding models to adequately compensate for this trajectory of residents.

### 4.3 End‐of‐life trajectories

Examination of specific end‐of‐life trajectories provides greater insight into the stages and diverse care needs of different life‐limiting conditions, which is an important reflection of the heterogeneity of those needing of palliative care.^19^, ^30^ Further, open and ongoing discussion about prognosis and illness trajectories is an integral component of palliative care, which helps to facilitate discussions around goals of care and advance care planning. As stated in the final report of the Royal Commission into Aged Care Quality and Safety, ‘knowledge about the course of a person's illness and the ability to pre‐empt or recognise and respond to changes in their condition is essential to providing good palliative care’ (p. 118).^31^ Our analysis indicated that over 60% of individuals in residential aged care were aligned to an organ failure end‐of‐life trajectory, which is often observed for people with conditions including heart failure, stroke, diabetes, end‐stage kidney diseases and chronic respiratory diseases. The frailty and dementia trajectory, which is typical for people with Alzheimer's and dementia, Parkinson's disease and multiple sclerosis, was slightly more prevalent than the cancer trajectory in residential aged care. However, people aligned to the non‐cancer trajectories are not as readily appraised as needing palliative care despite experiencing physical symptoms comparable to, or worse than, cancer patients.^32^ In 2019–2020 less than 2% of people with dementia in residential aged care were assessed as needing funding for palliative care, which was less than those who did not have dementia.^21^

### 4.4 Implications for care delivery

It has been reported that residential aged care staff often lack the expertise to recognise when a palliative approach to care is appropriate and generally adopt an acute life‐prolonging model of care unless admission documentation states otherwise.^33^ However, early delivery of palliative care reduces unnecessary hospital admissions and improves the confidence of aged care staff in discussing goals of care.^33^ A greater understanding of palliative care needs is essential to ensure adequate management of symptoms, medication review, reduced hospitalisation rates, advance care planning and ongoing communication around goals of care, and access to residential aged care nursing staff with expertise in palliative care, that is extended beyond older residents with cancer and those in the terminal phase of their disease. High‐quality palliative and end‐of‐life care should be a fundamental component of care in residential aged care, yet significant changes including models of care delivery, funding and workforce training are needed.^8^ In recognition of this need for improvement, the Australian Government in 2017–2018 announced $57.2 million over 6 years for the Comprehensive Palliative Care in Aged Care measure to support new and advanced ways at the provider, state and national level to improve palliative and end‐of‐life care for older people living in residential aged care.^34^ This was further supported in 2021 by Palliative Care Australia's eight‐point plan to improve palliative care in Australia's aged care sector, which includes for example, palliative care training for health and aged care workers in residential aged care, appropriate funding models and improved data relating to palliative care.^35^ Identification of palliative care needs is a vital first step in the planning, design and evaluation of such initiatives and as such, the population‐based estimate and characterisation of palliative care needs presented in this study may facilitate identification of best models of palliative care delivery in aged care.

### 4.5 Strengths and weaknesses

A major strength of our study is the use of population‐based linked aged care, health care and mortality data that captures all individuals who permanently entered residential aged care in three Australian states (comprising 70% of the residential aged care population) and died within the two‐year study period. Together with the use of multiple cause of death data, this provides a comprehensive overview of the potential palliative care needs within residential aged care and importantly quantifies the prevalence of different end‐of‐life trajectories to help inform the appropriate planning and delivery of palliative care in residential aged care. The inclusion of residents from three states is a limitation but these states account for 70% of Australia's residential aged care population. It is expected that the proportions of residents potentially benefiting from a palliative approach to care in other Australian states would be similar. A further limitation is that the population‐based approach adopted to estimate palliative care needs is subject to inaccuracies in the reporting of causes of death, which can be inconsistent across different disease groups.^36^ However, the use of multiple causes of death in the estimation of palliative care needs is expected to reduce any such errors. In addition, the estimate of palliative care needs does not indicate the timing or intensity of these needs and can only be obtained after death. Nevertheless, this estimate is useful for indicating the scale of palliative care needs within the residential aged care sector and for providing an improved understanding of those who might benefit from a palliative care approach.

## 5 CONCLUSIONS

There is extensive need for a palliative approach to care in residential aged care where most deaths result from a life‐limiting illness. This need is not reflected in the currently available funding‐based care needs assessment data and an alternative estimation approach is necessary to appropriately capture and characterise palliative care needs within the residential aged care sector. Palliative care, including end‐of‐life care, is a fundamental part of residential aged care service and care provision, which requires adequately trained multidisciplinary staff, appropriate resources and supports to deliver this care. Residents of aged care services should be able to expect equitable access to palliative care, regardless of demographic characteristics or diagnoses. This requires a broader view of palliative care that focusses not only on the last weeks of life but incorporates ongoing discussions about expected disease course and goals of care with residents and their families.

## FUNDING INFORMATION

This work is supported by funding the Australian Government's Medical Research Future Fund (MRFF) Primary Health Care Research Grant (MRFF1200056) and Rapid Applied Research Translation program (MRF9100005). Prof Inacio is supported by The Hospital Research Foundation Mid‐Career Fellowship (MCF‐27‐2019) and National Health and Medical Research Council (NHMRC) Investigator Grant (GNT119378). Dr Sluggett is supported by an NHMRC Investigator Grant (GNT2016277).

## CONFLICT OF INTEREST STATEMENT

No conflicts of interest declared.

## Supporting information


Table S1.

## Data Availability

Data are not available due to data privacy and ethical reasons.
