# Supplementary material for: Estimating potential palliative care needs for residential aged care: A population‐based retrospective cohort study
Source: Australas J Ageing. 2024 Jun 24;43(4):782–91. doi: 10.1111/ajag.13345 (PMC11671706; doi:10.1111/ajag.13345)
Supplement: Supplementary file 1 — Table S1. [file AJAG-43-782-s001.docx]

Supplementary Table 1: ICD-10-AM codes selected as ‘palliative care relevant’ by the French National Observatory on End-of-Life Care (ONFV)^1^ and their classification into end-of-life trajectories

| **End-of-life trajectory** | **Cause of death** | **ICD-10-AM Codes** |
| --- | --- | --- |
| **Cancer** | Malignant neoplasms | C00-C97 |
|  | Benign neoplasms | D00-D09, D32, D33, D37-D48 |
| **Organ failure** | HIV/AIDS | B20-B24 |
|  | Heart disease, including cerebrovascular disease | I231, I25, I27, I42, I50–I51, I60-I69, I70, I73-I74, I978 |
|  | Renal failure | N02-N05, N12, N18-N19, N25, N312, N319, N82 |
|  | Liver disease | K70-K77 |
|  | Respiratory disease | J40-J44, J47, J60-J62, J701, J80, J841, J96, J980-J984 |
|  | Diabetes | E10-E14 |
|  | Musculoskeletal system diseases | M05-M06, M13, M15, M360, M361, M13, M15, M21, M30-M54, M80, M81, M844, M86-M88 |
|  | Diseases of the blood | D61, D69, D70, D758, D86 |
|  | Diseases of the digestive system | K44, K50, K51, K55, K56, K85-K90 |
|  | Other | A523, A810, A812, B15-B19, E70-E72, E75-E77, E85, L40, L93, Q01-Q06, Q20-Q28, Q60-Q68, Q75-Q79, Q850, Q87-Q90, Q99, R060, R063-R068 |
| **Frailty and dementia** | Alzheimer’s, dementia, and other mental disorders | F01-F06, G30-G32 |
|  | Diseases of the nervous system | G03-G05, G07, G10-G12, G20-G23, G35-G37, G478, G518, G551, G608, G80-G83, G90-G99 |

ICD-10-AM: International Classification of Diseases, 10th Revision, Australian Modification

1. Observatoire National de la Fin de Vie (ONFV). Estimation du nombre de personnes nécessitant des soins palliatifs en France. In: Observatoire National de la Fin de Vie (ed.) Fin de vie, un premier état des lieux. Paris: ONFV, 2011, pp. 92–107.
